# Event and Comment

**Published:** 1920-11

**Authors:** 


### DENTAL REGISTER

THE MAGAZINE OF DENTISTRY

Vol. LXXIV NOVEMBER, 1920 No. 11

### EVENT AND COMMENT

**Adenoids and Tonsils as Related to Teeth** For many years dentists, and particularly orthodontists, have realized that nose and throat diseases are intimately related to dental affections. Recently we have stressed the facts that dental diseases are important influences in causing general systemic ill health. In fact, we have almost demonstrated that there is no such thing as a dental disease; that a disease in any part of the body may be a serious menace to the whole body. A most interesting clinic was made last summer in the Rochester Dental Dispensary, of Rochester, N. Y., which seems calculated to produce important and far-reaching results in the advancement of community hygiene.

The Rochester Dental Dispensary in the pursuit of data in the treatment of children's teeth of the public schools in the city of Rochester, noticed that a large number of the school children were suffering with nose and throat troubles which seemed to complicate dental treatments to such an extent that it seemed some preventive treatment was indicated. The dispensary authorities in a conference with the medical faculty and the city health officer decided that with the consent of the school board they would make a careful inspection of all the schools, giving particular attention to the nose and throat conditions. As a result of this inspection it was discovered that over 27,000 children are more or less seriously affected with diseased adenoids or tonsils, and that fully 9,000 of these children can not afford to have these properly treated.

As there were no hospitals prepared to treat so many children, and as there were no funds available, the Rochester Dental Dispensary placed its building and equipment at the disposal of the committee, and by the helps of the medical men of Rochester, 1,470 children were operated for tonsils or adenoids with successful results and not a single fatality. In view of the fact that the preliminary surveys indicated that eighty-seven per cent. of all the children in the city of Rochester needed such operations amply justify the experiment which the result has so unqualifiedly demonstrated. The experiment was scientifically and humanely executed and the outcome will be watched carefully, and we hope other experimenters will feel justified to repeat it until we shall be able to make it a rational and helpful method of eliminating another source of systemic infection.

Preventing medicine is gradually assuming a reality in practice which we have long hoped for, but which we have only blindly theorized about in most cases. If we can only keep the children's mouths and throats free from infection, at least while they are in early years of development, and can establish an adequate regime of hygiene, we may hope to grow normal human beings who will not fall by the wayside at every general epidemic that devastates our young and promising citizens.

Then think, too, of what this will accomplish for the prevention of dental caries and the almost universal condition of the irregularities of the teeth. It really looks like the beginning of the new and better day. We are giving a lot of notice to the report of this clinic, as it is very important and we cordially believe in it.

**The Tonsil-Adenoid Clinics** Twenty-seven thousand, seven hundred and forty-eight children in our city schools in need of tonsil-adenoid operations disclosed by survey. A startling condition, affecting not only the present health of the children of Rochester, but their future well-being, and constituting a serious menace to the whole community, has been revealed as a result of a survey made by the health authorities of the public schools. The astounding conclusions reached as a result of the investigation are summarized as follows:

That apparently 27,748 children attending the public schools of Rochester have enlarged and diseased tonsils and adenoids and should be operated upon if they are to develop physically and mentally as they should.

That 18,498 of these children presumably belong to families of limited means who can not afford to pay the fee charged by the regular practitioner for the tonsil-adenoid operation, and must be taken care of at clinics.

That 9,249 of these children belong to well-to-do families, able to pay the regular practitioner, and should be taken care of in regular practice.

CLINIC SHOWED NEED FOR SURVEY

The survey just concluded was a direct result of the recent intensive tonsil-adenoid clinic conducted by .the Rochester Dental Dispensary at which 1,470 children were operated upon during a period of seven weeks. The large number of children appearing for examination at that time and the fact that 2,256 operable cases were listed which could not be given the benefit of the operation, led Dr. George W. Goler, city health officer, to conduct a survey in the schools with a view to determining the number of children in Rochester actually afflicted with these disease-breeding growths.

The throats of 3,187 children attending School No. 20, School No. 23 and School No. 27 were examined by two physicians, one representing the Health Bureau and the other the Rochester Dental Dispensary, both agreeing in the diagnosis made.

### FACTS BROUGHT TO LIGHT

These examinations revealed the following facts:

That 2,792 children, or 87 per cent., needed operation for enlarged and diseased tonsils and adenoids.

That of the 2,792 children thus afflicted only 856, or 26 per cent., had been previously operated, and that 1,936, or 61 per cent., still require the operation.

That in the case of 776 children, or 24 per cent., immediate operation is not urgent; in the case of 762, or 24 per cent., operation is urgent, and in the case of 598, or 19 per cent., operation is very urgent. In all these cases, operation not only is desirable, but imperative, from the standpoint of the best physical and mental development of the child.

### EACH SCHOOL'S SHOWING

The tabulated report of the physicians follows:

SCHOOL No. 27

| | | |
|---|---|---|
| No tonsils apparent | 61 | 4.4% |
| Requiring no operation | 174 | 12.5 |
| Operation not urgent | 348 | 25. |
| Operation urgent | 412 | 29.6 |
| Operation very urgent | 213 | 15.3 |
| Operated previously | 183 | 13.2 |

SCHOOL No. 20

| | | |
|---|---|---|
| No tonsils apparent | 45 | 4.7% |
| Requiring no operation | 39 | 4. |
| Operation not urgent | 109 | 11.3 |
| Operation urgent | 199 | 20.6 |
| Operation very urgent | 242 | 25.1 |
| Operated previously | 331 | 34.3 |

SCHOOL No. 23

| | | |
|---|---|---|
| No tonsils apparent | 13 | 1.5% |
| Requiring no operation | 63 | 7.6 |
| Operaton not urgent | 119 | 14.3 |
| Operation urgent | 151 | 18.2 |
| Operation very urgent | 143 | 17.2 |
| Operated previously | 342 | 41.2 |

Selection was made of these three schools because the attendance is made up of children of all classes and it was believed is fairly representative of the school attendance of the whole city.

PERCENTAGE APPLIED TO CITY

Full significance of what is shown by these figures as to conditions existing in these three schools, as well as the alarming warning they convey, can not be appreciated unless they are considered in their relation to the situation in the city as a whole. Considered as truly reflecting actual conditions in all the public schools of the city, it was only necessary for the health authorities to ascertain that the total school registration for 1919, including the grade, parochial and orphanage schools, was 45,489, and to apply the percentage of children examined, to arrive at their startling estimate and justify the announcement that the health of 27,748 children of the city is impaired and their future development retarded by tonsils and adenoids.

It is pointed out that these figures include only children attending the grade schools, up to and including the eighth grade, all of whom presumably are under the age of sixteen years, and do not include the large number of children attending the high and junior high schools.

WELL-TO-DO FARE BEST

An analysis of the tabulated report of the physicians, it is pointed out, not only reveals an alarming condition in the large number of neglected children, but in addition leads to a conclusion that will be deplored by right-thinking citizens.

Reference to the figures for School No. 23, the attendance at which is made up largely of children of well-to-do families, shows that more than 41 per cent. of these pupils have had the benefit of operation, while in School No. 27, located in a less prosperous section of the city, the number of children who have been operated is only slightly over 13 per cent. In School No. 20, serving a neighborhood made up of both classes, the percentage of children operated is a fraction over 34 per cent.

These comparisons prove conclusively, it is pointed out, that more children of well-to-do families are being given the benefit of remedial and preventive surgery than are the children of families of limited means, and more of the latter are condemned to study and grow up under the handicap of physical defects because of the present lack in Rochester of clinical facilities to remedy those defects and place them on the road to better physical and mental development.

ADVICE BY PHYSICIANS

The result of the examination in School No. 23, it is pointed out, clearly disproves the impression held by a certain proportion of the public, that the removal of tonsils and adenoids is a fad and is not indorsed by the medical profession in general. More than 41 per cent. of the children attending that school have been operated, and the bulk of the work, if not all of it, has been done in private practice, by and on the advice of some of the leading physicians of the city.

It has long been recognized by the best physicians and medical authorities in all parts of the country that tonsils and adenoids should be removed early in the life of the child. They obstruct the nose and throat and furnish nests in which dangerous kinds of bacteria may grow, multiply and interfere with the growth, development and health of the body by poisoning it. When tonsils and adenoids persist they interfere with the growth and alter the shape of the face and cause the jaw to recede, the upper lip to protrude, the "hatchet-shaped" face, enlarged glands in the neck, distortions of the teeth in their settings, earache and decay of the teeth.

MORE LIABLE TO DISEASE

Children so affected are more likely to contract infectious diseases, such as whooping cough, which may lay the foundation for consumption; measles, which causes nearly 10 per cent. of the deafness in later life; scarlet fever, which causes much of the heart and kidney disease and many of the apoplexies after 60, and the joint affections or so-called rheumatism. Much of the backwardness of children in school, these physicians hold, is due to the presence of these growths. These are now known to be facts beyond question.

The alarming situation, as revealed by the survey, that 27,748 children of 16 years or under in Rochester need operation for the removal of tonsils and adenoids, is believed to be due to the following causes:

Ignorance or indifference of parents as to the injurious effects of glandular growths in the throat and the importance of having them removed as early in the life of the child as possible.

### WORK LET TO FALL BEHIND

Inability of hospitals to care for cases during the war and influenza epidemics, and failure since to conduct clinics for the purpose of catching up with accumulated cases; shortage of doctors on account of the war and the resultant decrease in number of cases operated in private practice.

Inability of families of limited means to pay the fee charged by the private practitioner.

The survey was made not only to gain a reasonably correct estimate of the true situation in the city, but with the determination to lay the facts, however serious and alarming they might be, before the public, with a view to stimulating action in several quarters that may result in the providing of ways and means adequately to meet the situation.

The conditions revealed by the survey are more grave than were expected; in fact, it is held that they constitute a serious menace of emergency proportions that in the interests of the health of the whole community should not longer be ignored.

### ECONOMIC SIDE IMPORTANT

It is pointed out by the health authorities that in considering the importance and necessity of giving children a fair chance to develop normally, both physically and mentally, which is the humane side of the question, the economic side also is important and should not be overlooked. The item of caring for the backward child and the repeater, due to loss of time on account of ailments caused by tonsils and adenoids, represents an enormous sum in money loss for schooling. The loss to parents, deprived of the services of older children also represents a large sum.

It is held that the task of caring for the 18,498 children who would have to be operated in clinics, is not, in view of the splendid equipment of the four public hospitals and the modern facilities of existing dispensaries, an insurmountable one. It is pointed out that the Rochester Dental Dispensary operated 1,470 cases in the short space of seven weeks. It is believed, also, that the clinics could take care of the 18,000 more cases and still furnish facilities to the physicians for the operation of the 9,000 or 10,000 that would have to be taken care of in private practice. Co-operation, it is held, would accomplish the task.

### CLINIC AN OBJECT LESSON

As to the success of a campaign to arouse the public to a greater appreciation of the necessities for and benefits of tonsil-adenoid removal, it is said that the success of the recent clinic at the Rochester Dental Dispensary, the fact that not a single casualty occurred, together with the great improvement already noted in a large number of children and reported by their parents, has more than blazed the way.

The deplorable situation revealed by the survey, it is pointed out, can not safely be ignored. The facts are believed to be beyond dispute, the consequences of continued neglect of the conditions shown to exist, as direful as they will be inevitable; therefore, it is held, the only question that remains to be considered is:

"What is going to be done about it, and who will do it?"

STEPS TO BE TAKEN

In the opinion of the health authorities it is imperative that the following steps should be taken immediately:

*First.* That clinics should be established with sufficient operating and nursing capacity to take care of within the next twelve months the more than 12,000 children of families of limited means urgently requiring the operation, a nominal fee only to be charged. The more than 6,000 other children of families of limited means not in urgent need of operation should be listed and held for observation so that they may be taken care of after the urgent cases have received treatment.

*Second.* That a city-wide campaign should be inaugurated, with the co-operation of the press, pulpit, school and health authorities, child welfare and other organizations, and citizens generally, in an endeavor to educate the public in the necessity and importance of having their children examined and, if treatment is needed, operated on. —Extracted from *Rochester Democrat,* October 18, 1920.

